# Impact of rotavirus vaccine introduction in Abidjan, Côte d’Ivoire

**DOI:** 10.1080/21645515.2022.2156231

**Published:** 2023-01-31

**Authors:** Alice Britoh Mlan, Rachel M. Burke, Hamidou Koné, Catherine Boni-Cisse, Rebecca N’Guessan, Flore Zaba, Lepri Nicaise Aka, Kofi N’Zue, San Koffi Adom, Sié Kabran Kouadio, Armel Bhérat Kouadio, Syndou Meité, Stephane Koffi, Hortense Faye-Kette, Keith Shaba, Bernard Ntsama, Joseph Biey, Negar Aliabadi, Jason M. Mwenda, Umesh D. Parashar, Jacqueline E. Tate

**Affiliations:** a Centre Hospitalier Universitaire de Yopougon, Abidjan, Côte d’Ivoire; b Viral Gastroenteritis Branch, Centers for Disease Control and Prevention, Atlanta, GA, USA; c Direction de Programme Elargi de Vaccination, Abidjan, Côte d’Ivoire; d Country Office for Côte d’Ivoire, World Health Organization, Abidjan, Côte d’Ivoire; e Institut Pasteur, Abidjan, Côte d’Ivoire; f World Health Organization Regional Office for Africa, Brazzaville, Republic of Congo; g World Health Organization Regional Office for Africa, Inter-Support Team for West Africa, Ouagadougou, Burkina Faso

**Keywords:** Rotavirus, pediatric gastroenteritis, acute gastroenteritis, rotavirus vaccine

## Abstract

Côte d’Ivoire introduced rotavirus vaccine in March 2017. Rotavirus surveillance is conducted at Centre Hospitalier Universitaire de Yopougon in Abidjan, the capital city. Children <5 years of age are enrolled in rotavirus surveillance if admitted to the hospital with acute gastroenteritis. We used sentinel surveillance data from 2014 through mid-2019 to compare trends in rotavirus pediatric gastroenteritis hospitalizations before and after rotavirus vaccine introduction. We used Poisson regression to analyze changes in rotavirus prevalence, adjusting for calendar month and accounting for total monthly admissions; January 2014 – December 2016 was considered “pre-vaccine,” and January 2017 – June 2019 was considered “post-vaccine.” Age distribution and severity were compared between periods using the Mann-Whitney U test. Rotavirus-positive admissions declined 51% (95% CI: 28%-67%), from 31.5% pre-vaccine to 14.9% afterward. The median age of rotavirus-positive children increased from 7 months (interquartile range [IQR]: 5–11) in the pre-vaccine period to 11 months (IQR: 7–18, *p* = .005) in the post-vaccine period. The median severity score decreased from 11 to 9 (*p* = .008) among all children, and from 12 pre- to 10.5 post-vaccine (*p* = .35) among rotavirus-positive children. Our findings suggest that rotavirus vaccine introduction contributed to reduced rotavirus hospitalization in Abidjan and possibly more broadly.

Rotavirus is a leading cause of severe pediatric gastroenteritis worldwide, with the highest burden in settings with high child mortality.^1,2^ In 2009, the World Health Organization (WHO) recommended rotavirus vaccines for inclusion in national immunization schedules globally.^3^ Since then, the impact of rotavirus vaccine introduction on reducing pediatric gastroenteritis hospitalizations has been widely observed, with the greatest absolute declines seen in countries with high child mortality.^4^

In Côte d’Ivoire, surveillance conducted in the capital city of Abidjan during 2000–2008 showed that approximately 30% of moderate to severe pediatric gastroenteritis was attributable to rotavirus.^5,6^ To reduce rotavirus burden, Côte d’Ivoire introduced RotaTeq (Merck), a pentavalent, live, oral rotavirus vaccine, into its national immunization schedule in March 2017, with three doses scheduled at 6, 10, and 14 weeks of age. Estimated complete-series coverage among children aged 12–23 months has increased year-over-year since introduction, but until recently lagged behind estimates for three-dose coverage of the pentavalent vaccine (diphtheria, tetanus, pertussis, hepatitis B, and *Haemophilus influenzae* type b).^7^ We used sentinel surveillance data from 2014 through mid-2019 to compare trends in rotavirus pediatric gastroenteritis hospitalizations before and after rotavirus vaccine introduction.

Côte d’Ivoire has conducted rotavirus surveillance as part of the WHO Global Rotavirus Surveillance Network since 2010.^8^ The sentinel surveillance site is the Centre Hospitalier Universitaire de Yopougon (CHU Yopougon), a tertiary-level hospital in Abidjan. Because surveillance faced several interruptions in 2010–2013, and because Côte d’Ivoire switched rotavirus vaccine products on a rolling basis beginning in April 2019, the analysis period is limited to January 2014 – June 2019.

Children <5 years of age were enrolled in rotavirus surveillance if admitted to the hospital with acute gastroenteritis (AGE), defined as ≥ 3 looser-than-normal stools within a 24-hour period with duration ≤14 days before admission. Hospital admission registers were reviewed daily for eligible children, and verbal informed consent was sought from the parents or guardians of children meeting eligibility criteria. Clinical and demographic data were collected from hospital records and parental report, using a standardized form in accordance with WHO Global Rotavirus Surveillance guidelines. Stool samples were collected by trained surveillance staff within 48 hours of admission and stored on site between 0 and 4C, in accordance with WHO Global Rotavirus Surveillance guidelines. Samples were transferred to the laboratory 2–3 times per week and analyzed for rotavirus using the Rotaclone_®_ enzyme immunoassay (EIA) kit. Tested samples were transferred to the WHO Regional Reference Laboratory in Accra, Ghana for long-term storage and quality control.

Data were stored and managed using Epi Info 3.5.1. A modified Vesikari score (range of possible values: 2–22) was used to quantify AGE severity based on clinical characteristics. We calculated rotavirus vaccine coverage among rotavirus-negative children eligible to have completed the rotavirus vaccine series (infants ≥4 months of age and born on or after 1 January 2017) and with vaccination status confirmed by vaccine card or clinic record. Rotavirus-negative children were used since rotavirus-positive children may be disproportionately unvaccinated, whereas rotavirus-negative children should better represent the underlying population and thus allow a less-biased estimate of rotavirus vaccine coverage. For analysis of changes in rotavirus prevalence, data were aggregated by month and year of admission, with January 2014 – December 2016 considered “pre-vaccine” and January 2017 – June 2019 considered “post-vaccine.” Poisson regression, adjusting for calendar month and accounting for total monthly admissions (using an offset term of the log of total monthly admissions), was used to estimate reductions in rotavirus positivity by year and calculate associated confidence intervals. To compare changes in age distribution and severity over time, we used the Mann-Whitney U test. For all comparisons, *p* < .05 was considered significant. Analyses were conducted using R version 4.0.5.

This activity was determined to be public health practice, and thus did not require review by an Institutional Review Board. Surveillance was conducted in accordance with all applicable local regulations.

During the analytic period (2014 – mid-2019), 534 children with AGE were enrolled in rotavirus surveillance at CHU Yopougon. All-cause and rotavirus-positive AGE admissions varied over time, but no clear seasonality was observed. Most (473/534; 89%) admissions were in children aged <24 months. Overall, 24% (128/534) of enrolled children tested positive for rotavirus.

Among 206 rotavirus-negative children enrolled after vaccine introduction, 109 had card-verified vaccination status. Within this group, 66 were enrolled when at least 4 months of age, of whom 89% (59/66) had received at least one dose and 55% (36/66) had received all three doses of rotavirus vaccine. Sample sizes were too small to assess differences by enrollment year or birth year.

Rotavirus prevalence among enrolled children declined markedly following vaccine introduction in 2017 (Figure 1). During the pre-vaccine period (2014–2016), 31.5% (92/200) of AGE admissions were rotavirus-positive, whereas in the post-vaccine period (2017–2019), only 14.9% (36/206) were rotavirus-positive (Table 1). This represents a decline of 51% (95% confidence interval [CI]: 28%-67%). Declines were larger among children aged <12 months: rotavirus positivity declined from 40.1% to 11.8%, a decrease of 69% (95% CI: 50%-82%).

**Figure 1.**
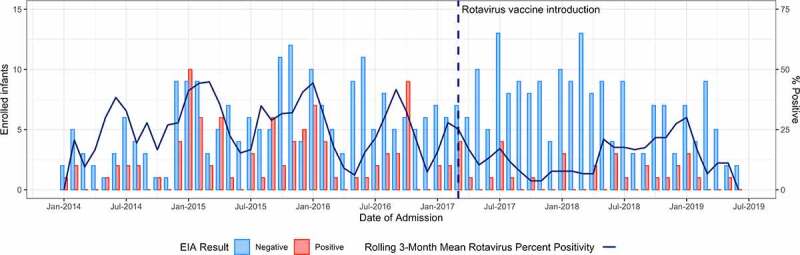
Children <5 years of age admitted with acute gastroenteritis before and after rotavirus vaccine introduction, by rotavirus EIA result.Abbreviations: EIA = Enzyme immunoassay.

**Table 1.** Rotavirus prevalence among children aged <5 years old enrolled in rotavirus surveillance by year and age group, and reductions over time.

Year	Total	Negative	Positive	% Positivity	% Reduction	95% CI*	P value*
*Children <5 years old*							
Pre-vaccine (2014–2016)	292	200	92	31.5	Ref	-	-
Post-vaccine (2017–2019)	242	206	36	14.9	51	(28, 67)	0.0004
2017	108	92	16	14.8	52	(19, 73)	0.008
2018	101	86	15	14.9	49	(14, 72)	0.017
2019	33	28	5	15.2	53	(−9, 84)	0.12
*Children <1 year old*							
Pre-vaccine (2014–2016)	182	109	73	40.1	Ref	-	-
Post-vaccine (2017–2019)	152	134	18	11.8	69	(50, 82)	<0.0001
2017	67	58	9	13.4	66	(36, 84)	0.002
2018	59	53	6	10.2	72	(40, 89)	0.003
2019	26	23	3	11.5	71	(18, 93)	0.042

*Calculated using Poisson regression controlling for month of admission, accounting for total admissions using an offset term in the model (log of total monthly admissions).

The characteristics of admitted children also changed following rotavirus vaccine introduction. Though the age of admitted children with all-cause AGE did not significantly change, the median age of rotavirus-positive children increased from 7 months (interquartile range [IQR]: 5–11) in the pre-vaccine period to 11 months (IQR: 7–18, *p* = .005) in the post-vaccine period. The median severity score decreased from 11 pre- to 9 post-vaccine (*p* = .008) among all children, and from 12 pre- to 10.5 post-vaccine (*p* = .35) among rotavirus-positive children.

Over the first 2.5 years following introduction of rotavirus vaccine in 2017 in Cote d’Ivoire, we observed a dramatic decline in the prevalence of rotavirus among children admitted for AGE in the sentinel surveillance hospital. Declines were greatest among children aged <12 months, showing a 69% decrease in rotavirus prevalence.

The present report provides the first evidence of rotavirus vaccine impact in Cote d’Ivoire, with results broadly similar to other findings from the region. In Burkina Faso, Bonkoungou et al. observed a 44% decline in overall positivity in the first two years following vaccine introduction, with a 54% decline observed in children aged <12 months.^9^ Similarly in Ghana, rotavirus positivity declined by 44% in the 2 years following vaccine introduction, with the greatest impact again in children aged <12 months.^10^ Surveillance data from Togo demonstrated a 37% decline in all-cause pediatric AGE hospitalizations, with a 52% decline among children aged <12 months, in the second year following rotavirus vaccine introduction.^11^ In our analysis, declines in rotavirus positivity were 51% overall and 69% among children aged <12 months, measured over the first 2.5 years after vaccine introduction. These trends are consistent with increases in estimated full-series rotavirus vaccine coverage, which grew from 40% in 2017 to 70% in 2019 ^7^. Though our estimated rotavirus vaccine coverage was only 55%, our sample was limited to children presenting to a single hospital during 2017 – mid-2019 and does not necessarily represent coverage among all Ivoirian children. Overall, our findings are consistent with global research demonstrating that rotavirus vaccine introduction is associated with reduced rotavirus morbidity.^4^

We also noted changes in the epidemiology of rotavirus cases. Median age increased significantly from 7 months in the pre-vaccine period to 11 months in the post vaccine period. An increase in the median age of rotavirus cases following vaccine introduction was also seen in Togo^11^ and elsewhere.^12^ This pattern may be explained by increasing proportions over time of rotavirus cases occurring among older children, who are less likely to be vaccinated as compared with younger children born after rotavirus vaccine introduction. We also observed a decrease in the severity of AGE, although this decrease was not significant among rotavirus-positive cases. Decreased severity of rotavirus and AGE cases is consistent with previous research from Malawi,^13^ and may be a mechanism for the decreases in AGE mortality often observed following rotavirus vaccine introduction.^4^

Inclusion of only a single surveillance site resulted in several limitations. First, the generalizability of the findings is limited, as patterns may be different in non-urban or non-academic hospital settings, or in other areas of the country. Second, the smaller sample size may have limited power to detect significant results in the last year of the analysis. Third, January and February of 2017 were included in the “post-vaccine” time period for analytic parsimony, despite the fact that vaccine was not introduced until March; however, sensitivity analyses (data not shown) demonstrated that this decision did not affect conclusions and had only minor effects on point estimates. Fourth, due to the relatively small number of samples that were genotyped, we are unable to make any presentation or conclusion on whether there were changes in circulating rotavirus genotypes; however, rotavirus vaccines are generally considered to provide protection across a variety of strains.^14^ Finally, because this is an ecologic analysis, we cannot make causal claims; however, we are not aware of any other possible explanations for declines in rotavirus positivity over the time period, outside of an effective vaccination program.

In this analysis of 4.5 years of surveillance data from a large academic hospital in Cote d’Ivoire, we found that rotavirus vaccine introduction was associated with a large decline in rotavirus prevalence among children hospitalized with AGE. Our findings suggest that rotavirus vaccine introduction likely contributed to reduced rotavirus burden in Cote d’Ivoire, consistent with the goals of the immunization program. Continued rotavirus surveillance will be important in Cote d’Ivoire to monitor any changes in epidemiology following the change in rotavirus vaccine product. Rotavirus surveillance, including genotypic surveillance, will also be informative in other geographies where rotavirus vaccine has been introduced.
